# Circulating Tumor DNA in Pediatric Cancer

**DOI:** 10.3389/fmolb.2022.885597

**Published:** 2022-05-12

**Authors:** Louise Doculara, Toby N. Trahair, Narges Bayat, Richard B. Lock

**Affiliations:** ^1^ Children’s Cancer Institute, Lowy Cancer Centre, UNSW Sydney, Sydney, NSW, Australia; ^2^ School of Women’s and Children’s Health, UNSW Sydney, Sydney, NSW, Australia; ^3^ University of New South Wales Centre for Childhood Cancer Research, UNSW Sydney, Sydney, NSW, Australia; ^4^ Kids Cancer Centre, Sydney Children’s Hospital, Randwick, NSW, Australia

**Keywords:** liquid biopsy, circulating tumor DNA, childhood cancer, NGS, ddPCR, minimal residual disease, relapse prediction, diagnosis

## Abstract

The measurement of circulating tumor DNA (ctDNA) has gained increasing prominence as a minimally invasive tool for the detection of cancer-specific markers in plasma. In adult cancers, ctDNA detection has shown value for disease-monitoring applications including tumor mutation profiling, risk stratification, relapse prediction, and treatment response evaluation. To date, there are ctDNA tests used as companion diagnostics for adult cancers and it is not understood why the same cannot be said about childhood cancer, despite the marked differences between adult and pediatric oncology. In this review, we discuss the current understanding of ctDNA as a disease monitoring biomarker in the context of pediatric malignancies, including the challenges associated with ctDNA detection in liquid biopsies. The data and conclusions from pediatric cancer studies of ctDNA are summarized, highlighting treatment response, disease monitoring and the detection of subclonal disease as applications of ctDNA. While the data from retrospective studies highlight the potential of ctDNA, large clinical trials are required for ctDNA analysis for routine clinical use in pediatric cancers. We outline the requirements for the standardization of ctDNA detection in pediatric cancers, including sample handling and reproducibility of results. With better understanding of the advantages and limitations of ctDNA and improved detection methods, ctDNA analysis may become the standard of care for patient monitoring in childhood cancers.

## Introduction

Pediatric cancer is the leading cause of disease-related death in children worldwide (Yeh et al., 2020). While advances in pediatric cancer treatment, international participation in clinical trials, risk stratification and supportive care have significantly improved the survival rates for childhood cancer, many patients will eventually relapse, reducing their chances of survival (Schultz et al., 2007; Pritchard-Jones et al., 2008; Northcott et al., 2011; Saarinen-Pihkala et al., 2012; Kremer et al., 2013; Hunger and Mullighan, 2015; Tierens et al., 2016; Steliarova-Foucher et al., 2017; Yeh et al., 2020). Thus, there is a critical need to safely and sensitively monitor disease progression, clonal evolution, tumor heterogeneity, development of resistance and relapse. The main cause of cancer-related death is relapse, which is known to arise from small numbers of drug-resistant minimal residual disease (MRD) cells throughout therapy at levels below morphologic detection (Brüggemann et al., 2012). Therefore, early detection of MRD is critical for evaluating treatment response, allowing the stratification of patients with high-risk disease to receive more intensive treatment regimens (Szczepański, 2007; Della Starza et al., 2019).

In hematological malignancies including acute lymphoblastic leukemia (ALL), the most common childhood malignancy, MRD cells are maintained in the bone marrow throughout chemotherapy and the level of MRD at the end of induction therapy is proportional to mortality and incidence of relapse (Pui and Campana, 2000; Sutton et al., 2009; Borowitz et al., 2015; Bartram et al., 2016). The detection of MRD via bone marrow biopsy has proven to be one of the strongest prognostic factors in the management of ALL in all disease phases for the last 2 decades (Paganin et al., 2008; Eckert et al., 2015). While MRD testing is not standardised in solid tumors, there are highly standardised and established guidelines for sample input and analysis in ALL (van der Velden et al., 2007). As such, MRD is the most appropriate model for novel molecular monitoring approaches. However, critical challenges with MRD testing have necessitated the need for non-invasive or minimally invasive methods for longitudinal monitoring of MRD as well as the molecular composition and characterization of a patient’s cancer.

In recent years, considerable technological advances have allowed for the detection of tumor-specific biomarkers in biofluids, e.g., plasma, including circulating microRNA/RNA (ctmiRNA/RNA), circulating tumor cells (CTCs), exosomes and circulating tumor DNA (ctDNA) (Ogawa et al., 2021). In particular, ctDNA detection has shown great promise for informing prognosis during treatment and at disease progression with potential for guiding clinical decisions and MRD monitoring (Oxnard et al., 2016; Khetrapal et al., 2018). The ability to interrogate the genetic landscape of cancers in real-time has fueled interest in translating the liquid biopsy into routine patient care. However, while there are promising studies on the prognostic value of ctDNA in adult cancers, there is little fundamental knowledge on its kinetics and prognostic value in pediatric cancers.

There are fundamental differences between childhood cancers and adult cancers (Kattner et al., 2019) and these may explain why pediatric cancer is a relatively untapped field for liquid biopsies. There are specific challenges in developing and implementing liquid biopsy in the clinic for pediatric cancers. In children with ALL, MRD negativity at Day 15 imparts an excellent prognosis, whereas in adult ALL, initial MRD assessment is performed at later timepoints, i.e., end of induction (Della Starza et al., 2019). This suggests that the kinetics of tumor clearance are dissimilar between children and adults, and this could potentially affect the timing and frequency of ctDNA testing. For clinical translation, the value of ctDNA must be demonstrated in each pediatric cancer and treatment program, therefore, a comparison of ctDNA with standard testing at hard endpoints is essential. Further, and at a genomic level, pediatric cancers generally harbor relatively few genetic mutations including gene fusions and chromosomal rearrangements, whereas adult cancers are more likely to exhibit numerous somatic mutations such as single nucleotide variants (SNVs) (Rahal et al., 2018).

The differences in genetic mutations are an important issue to consider, as the mutation to be detected will depend on the purpose of ctDNA testing, e.g., detecting treatment resistance to tyrosine kinase inhibitors for Philadelphia chromosome-like ALL, a high-risk subtype of pediatric ALL (Zhang et al., 2018). Current ctDNA assays have been used for the detection of somatic mutations such as EGFR (Jensen et al., 2021), however these somatic mutations have relatively lower frequencies in childhood cancer due to less exposure to mutagens including smoking and ionizing radiation. Also, liquid biopsy sampling for adult cancers is generally 5–20 ml of blood and in children, this amount is considerably lower, depending on age and weight (Kahana-Edwin et al., 2021a). It is probable that the sensitivity of ctDNA detection would be impacted by restrictions in pediatric sampling due to low levels of tumor burden in plasma. Furthermore, the sensitivity of bone marrow monitoring compared to peripheral blood is greater by 10-fold (van der Velden et al., 2002) and more work is required to confirm the complementary value of liquid biopsy markers such as ctDNA for disease monitoring.

In this review, we discuss the recent findings of ctDNA as a biomarker in the context of pediatric malignancies, including the challenges associated with detection in liquid biopsies. We examine ctDNA studies for risk stratification, treatment response and relapse prediction, highlighting the lack of fundamental knowledge of ctDNA in pediatric malignancies. We outline the requirements for the standardization of ctDNA detection in pediatric cancers, including sample handling and reproducibility of results. With better understanding of the advantages and limitations of ctDNA and the value of ctDNA for answering key clinical questions, ctDNA analysis may contribute to the standard of care for ongoing monitoring in childhood cancers.

## Challenges of Minimal Residual Disease Monitoring in Pediatric Cancer

In childhood ALL, the current gold standard for MRD monitoring involves bone marrow sampling followed by either flow cytometry analysis of cell-surface markers or real-time quantitative polymerase chain reaction (RQ-PCR) for the rapid detection of specific clonal rearrangements of immunoglobulin (Ig) and T cell receptor (TCR) genes, microdeletions, i.e., *IKZF1* or translocations (*BCR-ABL1* or *KMT2A*) (Burmeister et al., 2006; van der Velden et al., 2007; Venn et al., 2012; Hovorkova et al., 2017; Romano et al., 2019). However, there are limitations in the efficacy of contemporary MRD monitoring methodologies. Primarily, the sensitivity of current MRD testing techniques is limited by the number of cells (0.01% by flow cytometry) or amount of DNA (1.5 µg by RQ-PCR) processed from bone marrow (Othus et al., 2020). This limits the sensitivity to one ALL cell per 100,000 mononuclear cells (MNC) (10^–5^) (Della Starza et al., 2019). Achieving higher sensitivity of MRD detection, or any molecular monitoring methodology requires a greater amount of bone marrow sample which is not clinically feasible. Another limitation of current MRD techniques is target identification for MRD in other cancers. Recently, whole genome sequencing was used as an approach to overcome this limitation in a range of pediatric cancers (Subhash et al., 2021). Further, MRD detection in bone marrow alone cannot identify new leukemic clones that have potentially spread to extramedullary sites such as the central nervous system (CNS) or testes (Della Starza et al., 2019). Another consideration is that repeated bone marrow sampling is an invasive procedure, requiring general anesthesia in children.

Unlike hematological malignancies, MRD testing in solid tumors for risk stratification is not standardized, mostly due to the difficulties in sampling low concentrations of tumor cells in the blood (Chin et al., 2019). Detection of solid cancers usually requires a tissue biopsy paired with imaging techniques for confirmation. However, serial imaging can only detect macroscopic disease recurrence, thus lacking the sensitivity to inform molecular MRD or metastatic relapse (Chaudhuri et al., 2017; Luskin et al., 2018). Although several studies have demonstrated MRD detection using tumor-specific DNA as an alternative in neuroblastoma (Stutterheim et al., 2012; Hartomo et al., 2013; van Wezel et al., 2015), a recent review on studies that used RQ-PCR based MRD detection in this malignancy concluded that while the clinical significance of MRD in bone marrow or peripheral blood is still not clear in localized neuroblastoma, MRD markers were associated with poor outcome (Uemura et al., 2019) in metastatic disease. Whole genome sequencing performed by Subhas et al. identified tumor-specific DNA breakpoints as reliable targets for MRD detection in ALL, neuroblastoma and Ewing’s sarcoma, with detection of disseminated disease in both peripheral blood and bone marrow (Subhash et al., 2021). In brain tumors, the leading cause of cancer-related mortality in childhood, MRD detection relies on tumor resection which is challenging due to accessibility of the tumor site and limited quantity of tumor that can be surgically resected (Tang et al., 2020). Although alternative approaches to MRD monitoring in pediatric brain cancer including whole genome sequencing in medulloblastoma are rapidly emerging (Liu A. et al., 2021), prospective assessments are warranted.

It is important to note that MRD detection, i.e., the sampling of small numbers of cancer cells is inherently different from blood sampling and plasma-based ctDNA detection of disease. Therefore, while MRD analysis provides a standard model for liquid biopsy, these methods are measuring residual cancer cells from a single site versus circulating nucleotide biomarkers that represent an average of the total body tumor burden, depending on tumor type and phase of treatment. Given the challenges of diagnosis and detecting MRD in childhood cancer, liquid biopsy may provide significant advantages over conventional tissue biopsies including its use as an adjunct to disease detection and risk classification at diagnosis, assessing the quality of treatment response during treatment and providing an overall assessment of disease burden. Longitudinal monitoring of molecular composition and characterization of a patient’s tumor has further advocated for the use of liquid biopsy-derived markers in disease management, particularly ctDNA.

## The Biology of Circulating Tumor DNA

Total cell-free DNA (cfDNA) from normal cells is found at low levels (around 10–15 ng/ml) in all healthy individuals (Perkins et al., 2012). CtDNA is the tumor-derived fraction of total cfDNA (Underhill et al., 2016) and its levels vary greatly across different tumor types, with mutant allele frequencies (MAFs) or the percentage of mutant alleles as low as 0.01% of the total cfDNA (Haber and Velculescu, 2014). Hypothetically, the differences in ctDNA levels could be attributed to tumor type and treatment phase. While ctDNA is fragmented, its relative stability surpasses that of other liquid biopsy constituents including CTCs and cfRNA (Schwarzenbach et al., 2014).

Understanding ctDNA biology, size characteristics and mechanisms of release and clearance should be considered when developing sensitive detection techniques The available knowledge suggests two known processes for the release of ctDNA into the blood—passive release via necrosis or apoptosis, and active release or metabolic secretion *via* exosomes (Grabuschnig et al., 2020). Detailed descriptions of these mechanisms have been discussed in other reviews (Stroun et al., 2000; Jahr et al., 2001; Thierry et al., 2016). Reportedly, ctDNA fragments are frequently shorter than cfDNA derived from normal cells (Underhill et al., 2016; Hellwig et al., 2018; Mouliere et al., 2018; Liu Y. et al., 2021) and most recent evidence by Thierry et al. confirmed increased levels of fragmentation and nuclease activity in samples with high cancer cell ctDNA concentrations. The value of fragmentomics for distinguishing between cancer and normal cells is now clearer from these studies and there is potential for supplementing the molecular with the biological characteristics of ctDNA, as a strategy to improve cancer diagnostics.

Although ctDNA was initially thought to be released from the lysis of CTCs or micrometastases from the original tumor (Stroun et al., 2000), this was later disproved by Sorensen and colleagues, who showed that the high concentrations of ctDNA were not justified by detectable CTCs (Sorenson, 2000). The discrepancies between the number of CTCs and the amount of ctDNA could potentially be explained by the processes of ctDNA secretion, meaning that tumors with lower amounts of necrotic or apoptotic cells and without active mechanisms of exosome release may secrete low concentrations of ctDNA. The level of ctDNA shedding from these processes in childhood cancers has not been fully investigated and this could impact the sensitivity of any ctDNA detection method. CtDNA processing can be achieved by clearance from the liver, kidney and spleen, phagocytosis from macrophages, followed by nuclease degradation (Elazezy and Joosse, 2018). As the half-life of ctDNA is relatively short (16 min–2.5 h), it can be used to detect changes on the scale of hours, providing potential real-time analysis of tumor burden (Diehl et al., 2008). However, the half-life of ctDNA is proposed to be longer when it is bound to protein complexes or inside membrane vesicles, making it less susceptible to phagocytosis from tumor infiltrating cells (Khier and Lohan, 2018). This indicates that ctDNA, whilst being fragmented, is stable and protected from nuclease cleavage in the circulation. Therefore, ctDNA stability due to its nucleosomal structure may contribute to accurate quantification in serial follow-up sampling for disease monitoring. The molecular and biological characteristics of ctDNA have allowed for its detection with the rapid development of liquid biopsy techniques. However, ctDNA is still subjected to nuclease degradation and dilution by normal cfDNA, therefore processing and pre-analytical factors should be considered for accurate ctDNA quantification.

## Methods of Circulating Tumor DNA Detection

Recent technological advances have significantly addressed the challenges of ctDNA detection at low levels, however more work is required to understand the release of ctDNA and how this varies by the underlying cancer type. The methods for ctDNA detection are tailored to the clinical application and generally include: 1) single target PCR assays which are useful for disease monitoring; 2) small generic panels defined by the tumor subtype to allow for detecting subclonal markers which could potentially identify treatment resistance in addition to disease montoring; and 3) patient-specific panels using next generation sequencing (NGS) which allow for sensitive detection of many less common but clinically relevant mutations in multiple genes. The data obtained from sequencing the patient tumor can then be used for the design of patient-specific assays with reference to previous knowledge of the tumor type.

### Single-Target Assays Polymerase Chain Reaction

CtDNA analysis relies on the detection of somatic mutations that are not present in normal DNA. Therefore, prior knowledge of the genetic landscape of each patient’s underlying tumor is used for the detection through targeted assays, namely PCR methods (Forthun et al., 2019). Third generation PCR, also known as droplet digital PCR (ddPCR) has become increasingly popular in liquid biopsy for its ability to measure mutations, amplifications and deletions in DNA (Subhash et al., 2021). However, unlike traditional RQ-PCR methods, it does not rely on a standard curve for sample quantification (Taylor et al., 2017) and the DNA input is minimal. This is because ddPCR employs absolute quantitation and end-point measurement of lower concentrations of starting material sample input of 1 ng/reaction is possible with varying levels of multiplexing (Fitarelli-Kiehl et al., 2018) and Poisson statistics. In a study by Klega et al., ddPCR paired with NGS in pediatric Ewing’s sarcoma showed that ctDNA levels correlate with treatment response and disease-specific biomarkers (Klega et al., 2018) as ddPCR alone is not sufficient to identify all clinically relevant mutations and it is recommended to complement untargeted approaches (Andersson et al., 2021).

### Next Generation Sequencing-Based Methods

NGS is a valuable tool for identifying more comprehensive mutations across wider genomic regions (Kotrova et al., 2015). Currently, there are NGS protocols optimized for < 10 ng of cfDNA input, which is important as yields of cfDNA vary depending on the type of childhood cancer and tumour burden. It has been reported that the proportion of ctDNA to normal cfDNA can range from 0.01% to over 90% (Christensen et al., 2017). Moreover, NGS addresses the limitations of PCR methods, namely low throughput mutational profiling and the necessity for previous knowledge of ctDNA targets for detection (Shu et al., 2017). Sensitivities for NGS techniques depend on the depth of sequencing coverage and are expressed as percentage of MAF as the limit of detection. Most notably, molecular barcoding technology for ctDNA analysis has reduced error rate and improved sensitivity to ensure the valid consensus of sequences (Sato et al., 2019).

Knowledge of the original tumor is essential for serial disease monitoring and detection of resistance mutations with the potential for detection of subclonal disease. For the detection of low frequency clinically relevant somatic alterations (0.004%), Newman et al. used cancer-specific profiling by deep sequencing (CAPP-Seq) as an economical and ultrasensitive method with broad patient coverage (Newman et al., 2014). However, these novel assays are yet to be validated for clinical applicability. More recently, targeted NGS panels of known cancer mutations have shown to be useful for monitoring treatment response, with sensitivities in detecting mutations at MAFs of 0.1% (Gao et al., 2019).

The ability to sequence individual patient tumors is attractive in the field of personalized medicine as it is an unbiased approach for the detection of targets that were not previously associated with the individual’s tumor type. These methods typically involve sequencing of the patient’s tumor, from which the data are then used to design targeted assays, pending validated tumor-specific data. (Belic et al., 2016). With blind bias-corrected NGS analysis of NSCLC patient tumors, Paweletz et al. demonstrated detection of actionable driver and resistance mutations at 0.4% MAF. This assay detected mutations with no false positives, highlighting the potential of NGS ctDNA analysis for monitoring response and subclonal disease (Paweletz et al., 2016). Few studies have investigated NGS for high-throughput screening (HTS) for minimally invasive disease detection in childhood cancers (Applebaum et al., 2020; Peneder et al., 2021; Subhash et al., 2021). However, while significant progress has been made in validating tumor-specific NGS, more prospective and longitudinal ctDNA analyses from the timepoint of diagnosis will contribute to implementation of ctDNA for precision medicine.

In childhood cancers, where the ctDNA burden is expected to be different across underlying cancer types and where tumor burden is below 0.01% of total cfDNA, the available ctDNA is likely to contain less than one cancer genome (Fiala and Diamandis, 2018). Therefore, ultrasensitive NGS technologies will still depend on the absolute amount of ctDNA in every sample. Liang et al. showed that ctDNA detection via deep methylation sequencing paired with machine learning identified more patients with cancer compared to mutation sequencing analysis, highlighting detection of low tumor-derived signals (Liang et al., 2021). However, in another study by Lin et al., solid tissue-NGS detection of clinically relevant genomic aberrations and therapeutic targets was more reliable than plasma-NGS (Lin et al., 2021), thereby discouraging clinicians from making treatment decisions based on plasma-NGS alone. A previous limitation of high-throughput screening as a routine MRD diagnostic method in ALL is the tendency to amplify non-specific targets. However, in each MRD assay designed, background amplification is accounted for by the use pooled peripheral blood from healthy individuals (van der Velden et al., 2007), therefore, such controls are important for ctDNA assays. Although NGS technologies are providing a unique insight into ctDNA dynamics in cancer, the cost currently remains challenging for routine analysis (Gutowska-Ding et al., 2020). However, it is to be expected that these costs will decrease over time with increased depth of coverage as they did for traditional sequencing techniques, allowing this technology to be accessible for routine patient care (Chen and Zhao, 2019).

### Novel Methods

As solid tumor sampling and low levels of tumor burden are ongoing challenges for the assessment of disease in children with cancer, it is becoming more apparent that ultrasensitive and low-cost techniques for ctDNA detection can improve these limitations. Biosensing technology presents a unique alternative approach to conventional techniques of ctDNA detection, in part due to the lack of DNA amplification, that is, traditionally required for PCR methods. Biosensors are tools that produce a biological signal when detecting target analytes such as ctDNA, which is then converted into a quantifiable signal response (Wu et al., 2019). Electrochemical biosensors are more widely used for ctDNA detection due to their potential for clinical validity and commercialization due to portability and low cost. A review by Li et al. describes in detail all biosensor variations along with advantages, limitations and clinical values. Like PCR methods, biosensors can detect known aberrations, therefore an area for improvement is for biosensors to detect ctDNA in an unbiased manner at unknown mutated regions (Li et al., 2019).

### Circulating Tumor DNA Tests Approved for Clinical Use

Despite the challenges remaining to be overcome with liquid biopsy analysis, several commercial entities have developed ctDNA detection platforms. The most widely used liquid biopsy test is non-invasive prenatal testing (NIPT) which screens for fetal aneuploidy in pregnant individuals (Hartwig et al., 2017). To date, there are several FDA-approved liquid biopsy tests for ctDNA analysis on *EGFR* detection in NSCLC – the QIAGEN therascreen EGFR RGQ PCR kit and the cobas EGFR Mutation Test. These tests have been approved as companion diagnostics for solid tumor patients and have shown sensitivities of 0.81 and 3%, respectively, calculated as true positives and false negatives defined in accordance to the tissue-based test (Kwapisz, 2017). The QIAGEN kit is notable as a complementary screening test for patients with breast, NSCLC, colorectal and urothelial cancers, and detection of somatic mutations including EGFR, BRAF and KRAS in whole blood (Colomer et al., 2020). So far, it has been used to select patients in several Phase III clinical trials; however, more work is required to implement this in routine practice.

More recently, an NGS-based FoundationOne Liquid CDx test was approved as a companion diagnostic technology for mutations in NSCLC and prostate cancer to indicate eligibility for treatment (Kwapisz, 2017). Guardant360 CDx Liquid Biopsy was recently approved as another companion diagnostic for NGS detection of EGFR mutations which identified patients who would benefit from Osimertinib (Gupta et al., 2020). While NGS-based methods from the two tests could detect 300 and 60 genetic mutations, respectively, they could only cover a few genetic changes that match a patient to a specific therapy (Ignatiadis et al., 2021). Whether multi-gene panel assays for different cancers or targeted tests are superior for determining clinical outcome is yet to be confirmed. It is also noted that a negative result from these tests does not equate to negativity for the corresponding gene mutation, meaning that conventional tests should be performed in parallel (Ignatiadis et al., 2021). While preclinical studies of ctDNA have rapidly increased for adult cancer in recent years, implementation in routine disease monitoring pediatric oncology is yet to be established.

## Clinical Value of Circulating Tumor DNA Analysis for Pediatric Cancers

Prospective cohort studies for ctDNA analysis in adult cancers are currently conducted in the fields of diagnosis or risk stratification, monitoring response to chemotherapies and surgery and detection of treatment resistance or subclonal disease for solid cancers (Abbosh et al., 2017; Cohen et al., 2018; Kuwata et al., 2020; Lennon et al., 2020; Nakamura et al., 2020). These ctDNA studies have primarily focused on SNVs which are common in adult cancers. For example, the evaluation of ctDNA identified recurrent hotspot mutations in oncogenes such as *EGFR* and *KRAS,* both of which now have regulatory approval as companion diagnostics in Europe and the United States (Greig, 2016). For childhood malignancies, copy number alterations and chromosomal translocations are more prevalent (Gröbner et al., 2018) and these genetic alterations should be relied on for establishing methods of ctDNA detection in children with cancer.

Retrospective clinical studies of ctDNA in pediatric cancers have shown that ctDNA is detectable in various solid tumors at clinical timepoints on diagnosis, treatment duration and relapse (summarized in Table 1). With successful multi-centre trials, ctDNA assays may be implemented in pediatric oncology, as is the case for adult cancers. These trials should be designed to compare ctDNA with clinical response monitoring in the same population, in order to prove that ctDNA can inform clinical decisions, as previously shown by Viprey et al., who showed the prognostic value of mRNA MRD detection by comparison to conventional MRD techniques (Viprey et al., 2014). The current understanding of ctDNA for diagnosis and risk stratification in solid pediatric cancers is discussed below.

**TABLE 1:** Clinical ctDNA studies in pediatric cancer.

Cancer	Number of patients	Method of ctDNA detection	Target	Prognostic value	Reference(s)
Neuroblastoma	267	RQ-PCR	*MYCN*	Disease monitoring in patients with late-stage but not localized disease	Combaret et al., (2009)
Neuroblastoma	24	Microsatellite analysis (PCR)	11q loss	Treatment stratification	Yagyu et al., (2011)
Wilms tumor	120	Bi-sulfite sequencing	Differentially methylated regions (DMR)	Treatment stratification	Charlton et al., (2014)
Neuroblastoma	70	NGS	Copy number	Reflects tumor heterogeneity with potential for relapse prediction	Chicard et al., (2016)
Ewing sarcoma	20	ddPCR	EWSR1 fusions	Treatment response	Krumbholz et al., (2016)
Osteosarcoma	10	NGS	7 somatic aberrations	Potential for prognostic indication as ctDNA was detected before radiologic detection	Barris et al., (2018)
Osteosarcoma, neuroblastoma, Ewing sarcoma, Wilm’s tumor and alveolar rhabdomyosarcoma	46	NGS and ddPCR	Copy number variants and translocations	Treatment response and monitoring disease burden	Klega et al., (2018)
Ewing sarcoma and osteosarcoma	166	NGS	*STAG2* and *TP53* mutations, 8q gain	Potential for risk stratification	Shulman et al., (2018)
Diffuse intrinsic pontine glioma	15	NGS and ddPCR	H3K27M, *TP53*, *PDGFRA*, and *ATRX* mutations	Potential for guiding treatment decisions	Mueller et al., (2019)
Diffuse gliomas	85	NGS	*IDH1*, 1p/19q codeletion, *TP53*, *TERT*, *ATRX* mutations	Potential for disease monitoring	Miller et al., (2019)
CNS tumors	29	PCR	*BRAF* V600E	Potential for guiding treatment decisions	García-Romero et al., (2020)
Medulloblastoma	13	NGS and ddPCR	*TP53* and *PTCH1* mutations; *MYCN* and *GLI2* amplifications; *SUFU* deletions and 17p loss	Diagnosis and MRD detection	Escudero et al., (2020)
Neuroblastoma	11	NGS	*KMT2C*, *NOTCH1/2*, *CREBBP*, *ARID1A/B*, *ALK*, *FGFR1*, *FAT4 and CARD11*	Potential for treatment stratification	Cimmino et al., (2020)
Neuroblastoma	32	NGS	5-Hydroxymethylcytosine (5-hmC)	Treatment response	Applebaum et al., (2020)
Hepatoblastoma	3	ddPCR	*CTNNB1*	Treatment response	Kahana-Edwin et al., (2020)
Neuroblastoma	56	RQ-PCR	*RASSF1A*	Disease monitoring	van Zogchel et al., (2020)
Ewing sarcoma	20	ddPCR	EWSR1 fusions	Treatment response	Schmidkonz et al., (2020)
Diffuse midline glioma	10	ddPCR	H3.3K27M mutation	Potential for monitoring disease and treatment response	Li et al., (2021)
Ewing sarcoma and other pediatric sarcoma	126	NGS	ctDNA fragmentation	Disease monitoring and treatment response	Peneder et al., (2021)
Neuroblastoma	13	ddPCR	*MYCN*, *ALK* and segmental chromosomal aberrations	Risk stratification and diagnosis	Kahana-Edwin et al., (2021b)
Diffuse midline glioma	32	ddPCR	Hotspot driver mutations and single fusion events	Disease monitoring and treatment response	Izquierdo et al., (2021)
Ewing sarcoma, osteosarcoma, rhabdomyosarcoma and synovial sarcoma	17	NGS	Translocations and complex chromosomal rearrangements	Treatment response and early detection of relapse	Shah et al., (2021)

Myelocytomatosis viral oncogene neuroblastoma derived homolog (MYCN), Stromal antigen (STAG2), Tumor protein p53 (TP53), Lys-27-Met mutations in histone 3 (H3K27M), Platelet-derived growth factor receptor A (PDGFRA), ATP-dependent helicase (ATRX), Isocitrate dehydrogenase 1 (IDH1), Telomerase reverse transcriptase (TERT), Proto-oncogene B-Raf and v-Raf murine sarcoma viral oncogene homolog B (BRAF), Patched 1 (PTCH1), GLI family zinc finger 2 (GLI2), Lysine Methyltransferase 2C (KMT2C), Notch receptor 1/2 (NOTCH1/2), Cyclic adenosine monophosphate Response Element Binding protein Binding Protein (CREBBP), AT-rich interactive domain-containing protein 1A (ARID1A), Anaplastic lymphoma kinase (ALK), Fibroblast growth factor receptor 1 (FGFR1), FAT atypical cadherin 4 (FAT4), Caspase recruitment domain family member 11 (CARD11), Catenin beta 1 (CTNNB1), Ras association domain family member 1 (RASSF1).

### Neuroblastoma

At initial presentation, children with neuroblastoma typically present with a high tumor burden, therefore, it is no surprise that biomarkers are routinely used for diagnosis, prognosis and treatment response evaluation. In several studies, *MYCN* amplifications which are prevalent in neuroblastoma patients and are correlated with poor prognosis were detected in peripheral blood by qPCR (Combaret et al., 2002; Van Roy et al., 2017). In a study by Van Roy et al., copy number profiles showed strong concordance with conventional genomic hybridization from the primary tumor in plasma collected from neuroblastoma patients. NGS and ddPCR detection of *ALK* mutations associated with poor survival (Wang et al., 2013) demonstrated high sensitivity and specificity (Combaret et al., 2009). In a recent review by Van Paemel et al., all of these studies detected mutations in ctDNA that were previously not detected in the primary tumor, underlining the potential of ctDNA as a diagnostic tool for profiling tumor heterogeneity in neuroblastoma and indicating micrometastases (Van Paemel et al., 2020).

Whole-exome and targeted sequencing of tumor and plasma samples obtained at various timepoints were used by Chicard et al. to track SNVs and copy number variations in cfDNA collected from neuroblastoma (Chicard et al., 2016). The subclonal components of neuroblastomas, as well as the accumulation of genomic abnormalities (such as those in the MAPK pathway) in treatment-resistant disease, were investigated in this study. While the available evidence points to the importance of ctDNA in therapeutic decision-making and response evaluation, this is yet to be conclusive. For risk stratification, a prospective study of 13 patients revealed that ctDNA was a successful surrogate marker for molecular diagnosis of segmental chromosomal aberrations, MYCN amplifications and ALK variants, thereby presenting a relevant model for ctDNA analysis (Kahana-Edwin et al., 2021b).

### Brain Tumors

The amount of ctDNA that enters the blood is greatly limited by the blood-brain barrier (De Mattos-Arruda et al., 2015). To date, the presence of cfDNA in blood plasma has only been examined in pediatric patients with medulloblastomas, where it was detected in 40% of patients (Bettegowda et al., 2014). By analyzing only seven genes in cfDNA from cerebrospinal fluid (CSF), Martinez-Ricarte et al. were able to identify 17 of 20 patients (including two children) with diffuse gliomas (Martínez-Ricarte et al., 2018). Plasma from pediatric patients with diffuse midline gliomas was found to have tumor-derived histone H3 gene mutations (Huang et al., 2017). CSF, which has been shown by Seoane et al. to contain ctDNA to some level in adult patients (Seoane et al., 2019), provides another source of ctDNA for brain tumors. Since CSF is collected by lumbar puncture and routinely tested in children with brain tumors, ctDNA is yet to be fully investigated as a complementary marker for predicted CNS tumors. CtDNA in CSF was indicative of tumor response in two glioblastoma and two diffuse midline glioma patients (Izquierdo et al., 2021). More recently, MRD was detected in retrospective CSF samples from medulloblastoma patients who relapsed, earlier than disease progression detected by imaging techniques. The data from this study strongly support prospective analysis of ctDNA in future medulloblastoma clinical trials (Liu A. et al., 2021).

### Hematological Cancers

Plasma from 201 pediatric patients with diverse lymphoma subtypes (Mussolin et al., 2013) and 155 children with Hodgkin lymphoma (Primerano et al., 2016) had significantly higher cfDNA concentrations than plasma from healthy controls. Pathogenic *NPM-ALK* fusion genes were detected in plasma collected from patients with anaplastic large cell lymphoma by Mussolin et al. (Mussolin et al., 2013). In the same study, high cfDNA levels were concordant with poor prognosis in patients with Hodgkin lymphoma (Mussolin et al., 2013), and they were present at diagnosis in plasma from B cell non-Hodgkin lymphoma patients, but decreased after treatment (Machado et al., 2010).

Despite leukemia being the most common childhood cancer, ctDNA analyses have mainly been studied for pediatric solid tumors. In adult blood cancers, the evidence supports concordance between plasma cfDNA levels and disease progression in acute myeloid leukemia (AML) (Mueller et al., 2006; Fleischhacker and Schmidt, 2007; Jiang et al., 2012). Nakamura et al. demonstrated the prognostic value of ctDNA in AML, with ctDNA reflecting clonal dynamics in the bone marrow, predicting relapse and survival post-allogeneic stem cell transplant and showing concordance with MRD status (Nakamura et al., 2019). By strong correlation with conventional MRD testing in the bone marrow, the results suggest that ctDNA analysis in peripheral blood may eventually replace MRD monitoring in the bone marrow. Although this study was performed retrospectively, the evidence suggests the potential for prospective investigation. This potential was recently realized in a Phase 1b study involving 40 AML patients. The results showed that MAFs in plasma ctDNA were comparable to those in bone marrow and that lower ctDNA MAFs were an accurate marker of clinical response after the first cycle of treatment (Zeidan et al., 2020).

To date, ctDNA analyses in children with ALL have identified assays that are not sufficiently sensitive to inform prognosis (Schwarz et al., 2009; Cheng et al., 2013). However, ctDNA analysis could be used as a complementary test to bone marrow MRD measurement, as a representation of overall tumor burden in the patient (Cescon et al., 2020). In other pediatric hematological malignancies, studies have focused on the amount of total cfDNA as opposed to specific biomarkers in ctDNA (Mussolin et al., 2013; Primerano et al., 2016). While total cfDNA levels correlated significantly with treatment response and prognosis, they are not reliable as they lack the high specificity of ctDNA. Likewise, in children with different types of non-Hodgkin lymphoma, the total cfDNA levels were significantly higher than in healthy controls (Machado et al., 2010; Mussolin et al., 2013). In the plasma of patients with B-cell non-Hodgkin lymphoma, EBV-DNA was detected in the plasma of all EBV-positive patients. Plasma EBV levels decreased after treatment, then increased, suggesting tumor relapse (Machado et al., 2010). These findings show the potential of EBV as an alternative biomarker for ctDNA in children with EBV-positive tumors. For other pediatric blood cancers, further ctDNA studies are required to elucidate the value of ctDNA as an alternative biomarker in the clinic.

### Renal Tumors

A retrospective study by Jimenez et al. investigated plasma samples collected at the time of diagnosis of different types of kidney tumors in 18 patients. The results demonstrated sensitive detection of plasma tumor-specific copy number and/or single-nucleotide changes (Jiménez et al., 2019). As renal tumor biopsies are not routinely performed due to risk of rupture, genomic profiling of kidney tumor markers in the blood may be useful for guiding cancer-specific therapies (Van Paemel et al., 2020). Data from a prospective observational study of three patients showed that ctDNA in longitudinal samples could predict treatment response, however, the authors did not conclude whether ctDNA negativity was due to a fully resected disease or eradication of MRD (Kahana-Edwin et al., 2020). Therefore, further prospective studies that include patients who have relapsed or present with low levels of MRD are required to investigate the potential impact of ctDNA analysis for these clinical applications.

### Ewing Sarcoma

Rearrangements of the *EWSR1* gene, most typically *EWSR1-FLI1* and *EWSR1-ERG* rearrangements, are the diagnostic hallmark for Ewing sarcoma. The specificity of key fusion genes such as *EWSR1* allows for robust detection in ctDNA assays, however whether detection in blood can inform clinical decisions remains to be demonstrated. Recurring or increasing levels of the *EWSR1* fusion gene copy numbers were previously shown to be associated with relapse in 234 blood samples from 20 patients (Krumbholz et al., 2016). Patient-specific primers for ddPCR were developed in another study to detect tumor-specific *EWS-ETS* fusion gene breakpoint fragments in plasma samples from three Ewing sarcoma patients. In this study by Hayashi et al., plasma fusion gene fragments were detectable in two of these patients when the disease was radiographically undetectable, implying that monitoring tumor-specific *EWS-ETS* fusion gene breakpoint fragments in the blood could be a suitable personalized biomarker for early relapse (Hayashi et al., 2016). These studies highlight the notion that patients with Ewing sarcoma may benefit from therapy follow-up utilizing ctDNA for MRD analysis.

### Retinoblastoma

The vitreous fluid has been investigated retrospectively in 26 patients with retinoblastomas, even though the procedure is not minimally invasive or the site easily accessible. With shallow whole-genome sequencing, eye enucleation was predicted with tumor-specific copy number variations and *RB1* mutations discovered by Berry et al. in the vitreous fluid. In future trials, these biomarkers may guide the critical decision of whether to enucleate or rescue the eye (Berry et al., 2018). A recent prospective study by Xu et al. performed longitudinal molecular profiling ctDNA in aqueous humor samples, demonstrating clinical utility for monitoring treatment response (Xu et al., 2021). These conclusions were limited to small sample size, however, warrant further prospective validation.

## Challenges of Circulating Tumor DNA Analysis in Childhood Cancer

While clinical implementation of ctDNA detection tests for adult hematological and solid cancers has started to bear fruit, the same cannot be said for pediatric cancers. The general consensus is that ctDNA is a representation of tumor burden, however the link between ctDNA and CTCs needs further elucidation. The specific challenges of ctDNA analysis, including clinical relevance, standardization, sensitivity and units of measurement are discussed below.

### Clinical Relevance of Circulating Tumor DNA

It is important to understand the goal to be achieved with ctDNA analysis, in the context of diagnosis or risk stratification; treatment response monitoring or identification of treatment resistance or subclonal disease. The clinical question being asked would subsequently influence the method for ctDNA analysis, whether it be single-target assays, generic tumor panels or patient-specific panels. For example, whole exome sequencing analysis in 13 patients performed by Escudero et al. investigated common medulloblastoma germline and somatic driver mutations which could facilitate subtype classification and risk stratification. In the same patient cohort, ddPCR assays were valuable for MRD identification in CSF samples, while NGS revealed subclonal disease (Escudero et al., 2020). CtDNA could also be applied in other solid tumor treatment assessments where poor response is usually observed, such as end of induction therapy for high-risk neuroblastoma. In children with *ALK* mutant or *ALK* amplified high-risk neuroblastoma, ctDNA monitoring of this marker could identify patients who may benefit from alternative treatment strategies such as the ALK inhibitor lorlatinib, as demonstrated by Kahana-Edwin et al. (Kahana-Edwin et al., 2021b). There is strong evidence for the importance of profiling tumors at relapse, including RAS-MAPK pathway mutations known to occur in relapsed/refractory disease (Eleveld et al., 2015). Several pediatric studies have achieved this without prior knowledge of tumor-derived biomarkers in neuroblastoma (Cimmino et al., 2020) and more recently, in a panel of pediatric solid tumors (Stankunaite et al., 2021). These studies have shown the value of ctDNA for identifying potential mutations associated with drug resistance. While ctDNA has the potential for informing diagnosis, treatment response monitoring and identification of treatment resistance (Table 1), these studies were performed retrospectively in small cohorts. Prospective validation with comparison to standard-of-care at predefined timepoints are needed to translate ctDNA analysis into the clinical setting, where it could improve treatment response monitoring, detection of emerging resistance and MRD detection. However, for implementation into clinical care, ctDNA analyses require standardization.

### Standardization of Circulating Tumor DNA Analysis

There is currently no consistent method for liquid biopsy sampling, ctDNA extraction or analysis. A liquid biopsy workflow normally involves collection, biomarker isolation or detection and biomarker analysis, each of which requires procedure standardization. Compared to metastatic malignancies, localized cancers have reduced detectability of ctDNA (Bettegowda et al., 2014). In most solid tumor malignancies, ctDNA levels are < 10% in advanced metastatic disease and <1% in locally advanced non-metastatic disease (Bettegowda et al., 2014). Differences in ctDNA levels are attributed to the variability among patients with the same cancer and patients with different tumor types, stages, responses to treatment, tumor burden and cell replication (Bettegowda et al., 2014), all of which complicate ctDNA as a routine biomarker. Additionally, large volumes of plasma are required for sufficient amounts of ctDNA. Pantel et al. found that ctDNA concentrations in early-stage lung cancer patients can be as low as one genome equivalent in 5 ml of blood (Pantel, 2016). The age and weight of children are limitations that should be considered when collecting large volumes of blood for informative ctDNA data analysis. This is further confounded by the volume of sample, that is, clinically feasible to collect in children (Kahana-Edwin et al., 2021a). Detection and quantification of ctDNA therefore require highly optimal and sensitive methods which should be tailored for individual cancer types (Yi et al., 2017). This includes consideration of pre-analytical factors that affect the stability and yield of cfDNA.

There is no universal standard for pre-analytical procedures (sample collection and storage) and standard operating procedures (total cfDNA isolation and quantification) for ctDNA analysis. However, there are agreed conditions that have been shown to affect sample quality and stability of ctDNA. The impact of various cell-stabilizing blood collection tubes on cfDNA extraction was investigated by Alidousty et al., who demonstrated that cfDNA collection tubes containing preservatives stabilized total cfDNA and intact cells for up to 1–2 weeks at room temperature, which is more feasible for shipping, storage and batched processing protocols (Alidousty et al., 2017). This was contradictory to Parpart-Li et al., who showed cfDNA stability at 4°C (Parpart-Li et al., 2017). The optimal storage conditions for cfDNA sampling have not been standardised however, the general consensus is to store plasma at room temperature for a maximum of 4 h, followed by centrifugation at 2,500 × g at 4°C and long-term storage at low temperatures (Lommen et al., 2020).

Furthermore, lack of standardization for the type of sample (serum, plasma, urine and other bodily fluids) collected for ctDNA analysis is an issue often overlooked in many studies. However, studies performed by Thierry and Bronkhorst have since demonstrated that levels of ctDNA are much higher in plasma (Thierry et al., 2010; Bronkhorst et al., 2015). Lee et al. found higher cfDNA levels in serum and proposed that this occurred due to red blood cell clotting which then resulted in genomic DNA release, leading to dilution of ctDNA (Lee et al., 2001). Isolation of cfDNA for downstream analysis was investigated by Sorber et al., who concluded varying results in ctDNA recovery from pancreatic cancer plasma samples using a range of cfDNA isolation kits (Sorber et al., 2017). Limited yields of ctDNA may therefore affect assay sensitivity and specificity, regardless of whether they are broad coverage (e.g., NGS) or targeted detection (e.g., ddPCR). Thus, it is integral that methods for storage and processing of total cfDNA are optimal for sensitive downstream ctDNA analysis.

### Sensitivity of Circulating Tumor DNA Detection

The sensitivity of ctDNA detection techniques should always be considered for clinical validity in an MRD or early detection setting. For acute leukemias, MRD for treatment response assessment is most beneficial at early timepoints, when adequate numbers of tumor cells are present (Paganin et al., 2008). Sensitive ctDNA detection is especially challenging with low levels of tumor material, therefore, it is highly unlikely that ctDNA analyses can be used for early cancer diagnosis (Fiala and Diamandis, 2018). Sensitivities can be limited by the dilution of fragmented ctDNA with normal germline cfDNA (Sherwood et al., 2016). Andersson et al. discussed the potential for the lysis of normal blood cells, which may contaminate the sample (Andersson et al., 2021). Plasma samples must typically be centrifuged and separated within 1–4 h of collection. This alone presents with challenges for pre-analytical variability with cfDNA concentrations and purity with varying processing times (El Messaoudi et al., 2013). Comparison with genomic DNA from granulocytes was suggested to distinguish true mutations from underlying clonal hematopoiesis (Rossi et al., 2019), highlighting the importance of robust controls for ctDNA detection. Synthetic cfDNA may be useful as an external control to ensure consistent DNA input. There are cfDNA reference standards that are cell-line derived and commercially available in buffer or plasma with average lengths of 160-170bp. Specifically, these controls are fragmented DNA that mimics the fragmentation pattern of cfDNA (Zhang et al., 2017).

For current MRD measurement, routine analysis requires a threshold to determine the MRD status of the patient to determine the sensitivity and specificity of the MRD assay, which may be adjusted for every patient depending on the clinical question. For every cancer, excluding ALL, AML and chronic myeloid leukemia (CML), there is no gold standard for such a threshold. In the past, sensitivities were established by a serial dilution of cells in a sample from a healthy individual (Björklund et al., 2003) to determine the lowest number of cancerous cells that gives an MRD positive result. This has since been renamed the limit of detection (LOD) rather than sensitivity as sensitivity is defined by probability of a positive test result in a truly positive patient (Steinbach and Debatin, 2008). The threshold for negativity in ctDNA analysis is further complicated by uncertainty of undetectable tumor burden or robustness of the assay when there is limited material input. With a range of technologies available for ctDNA analysis in pediatric solid tumors: 1) the clinical value should take precedence; and 2) the sensitivity of novel tests should be compared to established methods for their translation into routine patient care.

### Unit Measurement of Circulating Tumor DNA

Guidelines for the most suitable unit of measurement for ctDNA have not been established and this is partly due to the purpose and type of ctDNA detection method. CtDNA concentration determined by ddPCR is expressed as ng/mL or haploid genome equivalents (copies)/mL. With NGS, Abbosh et al. measured genomic alterations in ctDNA as a fraction of total cfDNA (variant allele frequency or VAF) (Abbosh et al., 2017) whereas the studies previously described by Bettegowda et al. analyzed copies per volume of plasma (Bettegowda et al., 2014). The choice of technique for ctDNA analysis is confounded by the amount of input sample, which can be subject to intra- and inter-patient variability. A study on ctDNA in solid tumors by Bos et al. demonstrated better concordance between VAFs and mutant molecules per volume of plasma with ddPCR than NGS (Bos et al., 2021). However, the authors demonstrated that the variability between both units of measurement could have been partially explained by pre-analytical and analytical variables, as well as low and variable molecular coverage. To this end, appropriate sequencing quality controls to enable correction and better molecular coverage were suggested for better interpretation of ctDNA analyses.

In general, while targeted approaches were shown to have better analytical sensitivity than untargeted, both methods have been used extensively in clinical studies of pediatric cancer in adult cancers. They are required for: 1) investigating ctDNA biomarkers and their biological and clinical significance; and 2) designing personalized assays for ultrasensitive ctDNA detection. It is evident that standardization of units for ctDNA measurement in cancer, irrespective of adult or childhood, is challenging. Ultimately, the choice of ctDNA of biomarker and units of ctDNA analysis should always answer the clinical question at hand.

## Discussion and Future Insights

Recent liquid biopsy studies in childhood cancers have provided novel insights into the biology and dynamics of ctDNA. While the sensitivity and analysis of ctDNA measurement are improving, the clinical application of ctDNA is yet to inform prognosis and evaluate treatment response in pediatric patients. CtDNA applications for pediatric cancers are not as advanced as in adult cancers and the studies summarized above are retrospective studies in small cohorts. Despite the challenges of ctDNA analysis, these studies have shown the potential for ctDNA tests in pediatric tumors with high turnover of immature cancer cells. Furthermore, there were previously limited clinically relevant experimental models to mimic childhood cancer, relapse and treatment resistance, however this has significantly improved with patient-derived models now used in large translational programs in pediatric cancer (Janssen et al., 2017; Rokita et al., 2019; van Tilburg et al., 2021). There is a definite need for prospective trials at multiple sites to fully investigate the value of ctDNA, especially with most children with cancer enrolled in randomized control trials. These studies may strengthen the significance of ctDNA in risk stratification, therapeutic response, relapse prediction and MRD monitoring.

The dynamics of ctDNA in the context of MRD or clinically-defined disease stages are poorly understood. For clinical implementation, robust guidelines for standardised sample collection and processing, as well as correlation to clinically significant outcomes in large clinical trials are needed. Comparisons between ctDNA analysis and current standard of care response assessments such as conventional MRD monitoring in ALL, are also necessary to understand the value that ctDNA provides. To our knowledge, there are few studies that investigate ctDNA for cancer screening or early detection, and the available data show ctDNA assays with low sensitivities for early cancer diagnosis. With cfDNA prenatal testing for certain fetal chromosomal abnormalities now standardized for pregnant individuals, identification of high-risk mutations for cancers for cancer predisposition syndromes is a potential application of ctDNA however, this will need to be validated in prospective studies. Different approaches to ctDNA detection have recently been developed, with promising technology in the field of nanomedicine for ultrasensitive ctDNA detection without the need for DNA amplification. For sensitive ctDNA assays to succeed as disease monitoring tools, they must demonstrate strong value in improving patient care as is the case for regulatory approval of novel drugs. CtDNA in combination with other liquid biopsy constituents including CTCs and exosomal miRNA may better reflect the cancer in one blood sample as accurately as possible. Information from all these samples may be worthwhile in providing a more detailed comparison of the genomic alterations present in ctDNA. The overarching challenge is to develop a liquid biopsy test, that is, rapid, patient-specific, low cost, clinically translatable and applicable to a wide range of pediatric cancer patients.
